# Genomic barcoding for clonal diversity monitoring and control in cell-based complex antibody production

**DOI:** 10.1038/s41598-024-65323-7

**Published:** 2024-06-25

**Authors:** Niels Bauer, Christoph Oberist, Michaela Poth, Julian Stingele, Oliver Popp, Simon Ausländer

**Affiliations:** 1 Large Molecule Research, Roche Pharma Research and Early Development (pRED), Roche Innovation Center Munich, Penzberg, Germany; 2 Gene Center and Department of Biochemistry, Ludwig-Maximilians-Universität München, 81377 Munich, Germany

**Keywords:** CHO, Barcoding, Lineage tracing, Antibody production, Biotechnology, Biologics, Expression systems, Molecular engineering

## Abstract

Engineered mammalian cells are key for biotechnology by enabling broad applications ranging from in vitro model systems to therapeutic biofactories. Engineered cell lines exist as a population containing sub-lineages of cell clones that exhibit substantial genetic and phenotypic heterogeneity. There is still a limited understanding of the source of this inter-clonal heterogeneity as well as its implications for biotechnological applications. Here, we developed a genomic barcoding strategy for a targeted integration (TI)-based CHO antibody producer cell line development process. This technology provided novel insights about clone diversity during stable cell line selection on pool level, enabled an imaging-independent monoclonality assessment after single cell cloning, and eventually improved hit-picking of antibody producer clones by monitoring of cellular lineages during the cell line development (CLD) process. Specifically, we observed that CHO producer pools generated by TI of two plasmids at a single genomic site displayed a low diversity (< 0.1% RMCE efficiency), which further depends on the expressed molecules, and underwent rapid population skewing towards dominant clones during routine cultivation. Clonal cell lines from one individual TI event demonstrated a significantly lower variance regarding production-relevant and phenotypic parameters as compared to cell lines from distinct TI events. This implies that the observed cellular diversity lies within pre-existing cell-intrinsic factors and that the majority of clonal variation did not develop during the CLD process, especially during single cell cloning. Using cellular barcodes as a proxy for cellular diversity, we improved our CLD screening workflow and enriched diversity of production-relevant parameters substantially. This work, by enabling clonal diversity monitoring and control, paves the way for an economically valuable and data-driven CLD process.

## Introduction

Recombinant antibodies continue to lead biopharmaceuticals in numbers of approvals (53.5% of US and EU approvals 2018–2022), sales (80.2% of total biopharmaceutical sales) and their impact on global health^1^. 67% of recombinant antibodies are produced by mammalian cell systems^1^, dictated by the need of correctly folded and glycosylated protein with human-like post-translational modifications (PTMs).

All cells used in a mammalian expression system, including Chinese hamster ovary (CHO), mouse myeloma line (NS0), and HEK293 cells, have been initially isolated from living tissue^2^. During the immortalization process each of these cell lines have undergone undefined selective expansion of sub-lineages, exhibiting substantial genetic and phenotypic heterogeneity^3^. As such, mammalian expression systems demonstrate close resemblance to cancer cells, when comparing genetic and phenotypic instability observed within cancer patients or in bioreactors^3^.

The majority of mammalian expression systems use random integration and/or gene amplification systems based on dihydrofolate (DHFR) reductase or glutamine synthetase (GS), resulting in further increased intrinsic heterogeneity of such expression cells^4^. Gene amplification procedures aim to boost transgene copy number dramatically (up to 1000 copies per cell) by using either DHFR, or GS-deficient CHO cell lines for the transfection, followed by gene amplification in the presence of methotrexate (MTX) or methionine sulphoximine (MSX), respectively^5^. These procedures result in substantial heterogeneity due to copy number variation, rearrangement of transgene cassettes, and position effects of the integrated plasmids^6,7^. The final total variety in cellular behavior enables screening of genetic and phenotypically distinct cell lines with high likelihood to identify high producer cells.

The intrinsic cellular heterogeneity of expression systems is in stark contrast to regulatory quality control requirements (*i.e.* Quality by Design), which aim to reduce product heterogeneity to a minimum. To ensure a robust and reproducible production process, cellular heterogeneity needs to be limited after generation of a suitable expression pool. Current regulatory guidelines therefore require that the producing cell is being derived from a single cell origin, as clonal derivation is generally believed to increase the likelihood of stable product quality^8–10^.

This regulatory view on the importance of clonal derivation was affirmed recently^11^, despite increasing evidence that “clonality” itself is unsuitable to address process robustness or reproducibility during manufacturing^12^. Rather clonal steps display a “genetic bottleneck” in which genomic and phenotypic distinct populations are separated briefly until giving rise to emerging new populations^13–15^. Remarkably, even clonally derived cell banks can give rise to genetically distinct subpopulation within less than 2 months^16^. Thus, clonal cell lines still display a wide array of production-relevant phenotypes.

Despite many studies describing the types of genetic and phenotypic variability within mammalian expression systems, the underlying sources remain incompletely understood^14,17,18^. Previous studies hint towards an interplay between genomic plasticity, epigenetics, stochastic gene expression, changing environmental conditions, copy number and positions effects^19,20^. While most of these areas remain unsolved, the field has increasingly moved to site-specific integration technologies that enable exclusion of the copy number and position effects of transgenes. This has resulted in increased process stability and displays the most promising approach to compromise between clonal variability and process stability so far^21,22^.

In the development of biopharmaceutical-producing cell lines, the lack of insight into cellular biology prevents an economic and data-driven cell line development (CLD) process. As variation within a given cellular population and their influencing factors remain elusive, excessive clone screening is required. Especially, it is unclear to which extent clonal variability is inherently occurring and which part is induced by changes in the environmental conditions defined by different CLD stages.

DNA-based barcoding of cells has emerged as a powerful technology with broad applications in basic biology and synthetic biology. Barcoding single cells in vivo allows for tracking their fate in diseases and reveals novel insights in genotypic and phenotypic profiles of *e.g.* cancer sub-lineages^23,24^. Pooled knock-in screenings of genetically-engineered barcoded libraries enable high-throughput testing of millions of genetic variants in an isogenic context. Consequently, massive parallel phenotypic perturbation screenings that are coupled to next-generation sequencing readouts in bulk or at single-cell level become feasible^25^. Recently, genetically-barcoded knock-in libraries have been used for deciphering optimal targeted integration loci in CHO antibody producer cells^26^ as well as first genome-wide pooled CRISPR KO screenings to improve cellular bioproduction properties^27^.

Here, we have further expanded the application area of genetic barcoding and developed a cellular single-copy targeted integration barcoding strategy based on dual-plasmid recombinase-mediates-cassette exchange (RMCE)^22^ to monitor CHO producer cell lineages expressing three distinct complex bispecific antibodies. This enabled quantification of clonal diversity at pool level as well as clonal lineage tracing during selection, single cell cloning, expansion, and subsequent testing in scale-down bioreactors.

Using this system, we could quantify, for the first time, the absolute number of integration events generated by dual plasmid RMCE, which revealed stable pool composition pre-/ and post-selection. We demonstrate that very few cells (less than 0.1% of the original population) successfully undergo dual plasmid RMCE and simultaneously survive selection pressure, and discovered that dominant clones rapidly overgrow the population during routine cultivation. By discriminating between cell lineages within stable pools, we establish that the clonal origin largely determines phenotypic variability regarding production-relevant parameters, which further correlates with shared epigenetic profiles. In the context of targeted integration (TI), we introduce cellular diversity as a constant feature, largely independent of environmental influences during the CLD process. We demonstrate that cellular barcodes can be used as a proxy for cellular diversity, resulting in an improved CLD screening workflow and substantially enriched diversity of production-relevant parameters. Collectively, these data highlight the use of genomic barcoding as a key method to monitor and control cellular phenotypes during TI-based CLD workflows.

## Results

### Low transcriptome diversity within cell line development workflow

**Figure 1:**
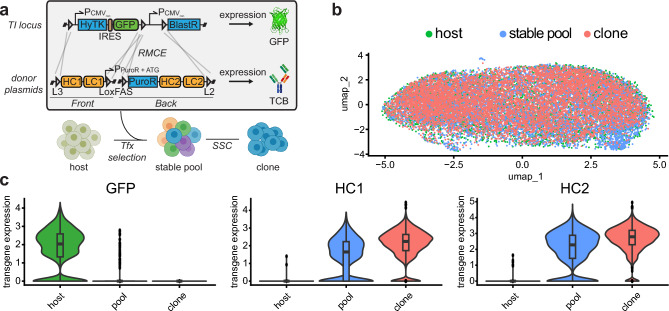
CHO cells are remarkably similar across the CLD process (**a**) Dual plasmid (front and back plasmid) integration strategy via recombinase-mediated-cassette-exchange (RMCE) into a CHO host cell line containing a landing pad (with lox acceptor sites: L3, LoxFAS, L2). Host cells express GFP and sensitive to FIAU due to thymidine kinase expression. Single copy targeted integration is enforced by negative selection (FIAU) and positive selection (puromycin). The start codon for the puromycin CDS is located on the front plasmid. (**b**) Overlay of single-cell transcriptome profiles from host cells containing the RMCE landing pad (expressing GFP), a stable expression pool, and clonal cell line originating from the expression pool. (**c**) Average levels of transgene expression in single-cell transcriptome profiles of host, stable pool, and clonal cells. UMAP, uniform manifold approximation and projection; HCL, host cell line; Pool, stable expression pool; Clone, stable expression clonal cell line.

We were interested in the cellular population diversity at different stages of an isogenic TI CLD platform^22^. This platform is based on simultaneous dual-plasmid RMCE-mediated targeted integration into a single genomic locus thus generating isogenic cells, which theoretically excludes variability derived from position effects, copy number and epigenetic silencing (Fig. 1a).

We harvested cells at three different stages of the CLD process: the GFP-expressing host cell (“host”), a stable bispecific antibody expression pool with a distinct gene configuration in our TI platform (“stable pool”), and a final producer clone (“clone”) that has been derived from the same stable pool. Each population was transcriptionally profiled on single-cell level using scRNAseq (Chromium Single Cell 3’ solution) and, after merging and batch correction, we observed no relevant cell population substructures (Fig. 1b). We hypothesized that variability in the expression of genes encoding the recombinant protein is insufficient to contribute to significant global transcriptomic differences during the CLD process.

We therefore specifically analyzed GFP expression within the host population and noticed some degree of variability with a fraction of cells showing no GFP expression (Fig. 1c, left panel). This variability was more pronounced in stable pools where a substantial fraction of cells showed no detectable expression of heavy chain 1 (HC1) and 2 (HC2, Fig. 1c, middle and right panel). In contrast, we observed a substantially smaller variability in HC1 and HC2 expression in a monoclonal cell population. This population had underwent recent single cell cloning and had been pre-selected based on high production performance.

The data indicate that while the overall cellular gene expression is remarkably similar across transfection, stable pool selection, and single cell cloning, transgene expression remains variable in host cells and stable expression pools.

### Single-copy targeted barcode integration in stable CHO producer cells

To shed light on the source of transgene expression variety we aimed to implement a genetic lineage tracing method within our CLD workflow. To achieve this, we developed an exhaustive single-copy genetic barcode labeling method, implemented within a state-of-the-art CLD workflow applicable for therapeutic protein production. Most barcode delivery methods (retroviral-based) lead to an inhomogeneous labeling of the population with possibly no or multiple barcode integration per individual cell clone. In contrast, the implementation of a barcode within an isogenic dual-plasmid RMCE-mediated targeted integration into a single genomic locus, allows for the selective expansion of clones with mainly single-copy integration^22^. Notably, the start codon of the puromycin resistance gene is placed on the “Front” expression vector, ensuring that only cells with in-frame- and targeted-integration survive the selection procedure. Additionally, all cells with off-target integration of the expression plasmids do not lose the Thymidine kinase selection marker that is encoded in the landing pad of the host cell line. Overall, only clonal cells undergoing correct on-target recombination between the three LoxP sites become resistant to puromycin and survive in the presence of FIAU. This stringent selection process substantially increases the proportion of single-copy targeted integration survivors.

**Figure 2:**
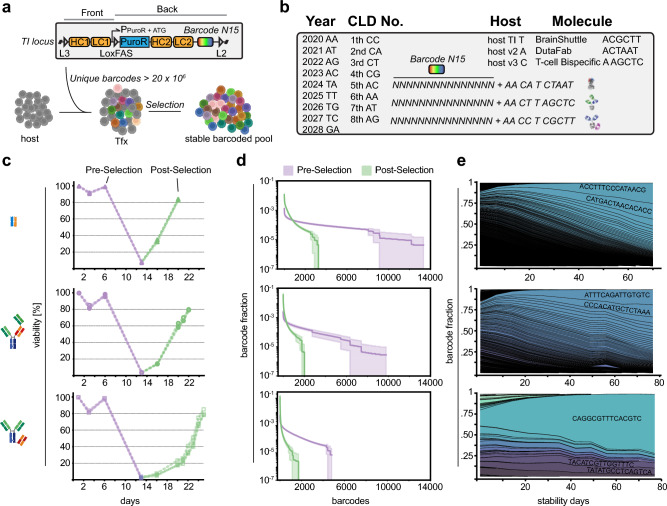
Single-copy targeted barcode integration in stable CHO producer pools (**a**) Notably, the barcode library (N15) is placed adjacent to the L2 lox site to discriminate on- and off-target integration events. (**b**) Barcode sequence design used in this project. Total barcodes combines a randomized N15 region with 10 fixed positions for cell line meta-data. (**c**) CHO host cells were transfected with Front and Back plasmid containing the barcode library at day 0. Selection started at day 6 and continued until cell pool recovery (cell viability > 80%). Note the different cell pool recovery kinetics. (n = 3) (**d**) Barcodes reflect the amount of successful RMCE events and thus the pool diversity. Note the substantially lower pool diversity at the post-selection time point (green) as compared to the pre-selection time point (purple). The error bands represent the standard deviations of biological replicates (n = 3) (**e**) Pool composition drifts during prolonged cultivation and diversity decreases substantially within 80 days. Notably, the effect is more pronounced in case the initial pool diversity is lower. Averaged fraction values of biological replicates (n = 3). (**f**) Retrospective analysis of barcode composition found after single cell cloning and random sampling for 96 clones (ambr15). Width of line indicates relative fraction of cells carrying a unique barcode in the cellular population.

A N15 barcode region was included into one of two plasmids (“Back”) adjacent to the lox site outside of the coding sequence (Fig. 2a). The N15 region is placed in close proximity to the genomic area outside the landing pad, allowing discrimination between on-target and off-target integration events by positioning of the primer binding sites during amplicon deep sequencing. To additionally incorporate cell line metadata, we added 10 fixed positions to the N15 region and devised a nucleotide representation of year, number of CLD (in the respective year), used host cell line and expressed biotherapeutic molecule (Fig. 2b). We validated the plasmid library by amplicon deep sequencing and observed a near uniform barcode representation with homogenous nucleotide composition at each position (Supplementary Fig. 1a,b). This provides a minimum diversity of > 2 × 10^7^, enough to label 10^5^ cells with < 0.3% collision probability (Supplementary Fig. 1c,d).

To investigate a representative repertoire of therapeutic proteins produced in CHO cell lines, we selected three different molecules based on the observed viability loss of the cell population during stable pool selection: 5–10%: **M1** DutaFab, 1–5%: **M2** TCB, < 1%: **M3** BS-Fusion (Fig. 2c). We analyzed the clonal diversity of respective CHO cell pools expressing M1-M3 during stable pool selection at two time points: (i) pre-selection at day five post-transfection and (ii) post-selection at the day the cell population reached ~ 80%viability.

We transfected 4.5 × 10^6^ cells of the host cell line with respective antibody-encoding TI and Cre recombinase-encoding plasmids by electroporation at day 0 followed by a recovery phase without selection pressure. Selection pressure (+ Puromycin & FIAU) was started subsequently at day 5 (“Pre-Selection”) and lowest cell viability was reached at day 13 for all CHO pools. The recovery time until reaching ~ 80% cell viability differed dramatically depending on the complexity of the encoded molecule and associated gene configuration (M1: day 20, M2: day 22, M3: day 26) (Fig. 2c). This observation was consistent with our previous experience showing that the speed of CHO pool recovery during resistance marker-based stable cell pool selection is linked to the complexity of molecules encoded on the expression plasmids (unpublished observation). DutaFab (M1) expressing cell lines recover quickly, potentially because of their overall smaller size and corresponding smaller plasmid sizes. In contrast, TCBs (M2) and BS-Fusion (M3) molecules are complex multi-domain fusion molecules, which makes them increasingly difficult-to-express for CHO biofactories^28^. Interestingly, pool composition was approximately 3.5–4.0 times higher at the pre-selection time point (M1: Ø 10060, M2: Ø 6355, M3: Ø 4560) as compared to post-selection across molecules, indicative of rapid clone loss during the stringent selection process (Fig. 2d). Recovered stable pools consisted of a low total amount of barcodes (M1: Ø 2884, M2: Ø 1691, M3: Ø 1158) with a skewed population distribution already at post-selection. Notably, in M3 the most abundant barcode encompassed 10% of the population at the post-selection time point.

Next, we analyzed population dynamics of the three M1-M3-expressing stable CHO pools for a total of 11 weeks with selection pressure. In all three biological replicates, the number of barcodes detected in each population decreased substantially with loss of 80–87% of barcode variants over the observed time course (Fig. 2e). This indicates that stable CHO pools display rapid clonal dynamics under standardized cell cultivation conditions.

Overall, these experiments demonstrate that CHO producer pools generated by TI display a low diversity, which further depends on the expressed molecules, and undergo rapid population skewing towards dominant clones.

### Improving efficiency of limited dilution and alternative proof-of-monoclonality by genetic barcoding

Motivated by the success of using cellular barcoding for monitoring CHO producer pools, we next explored the use of barcoding for assurance of monoclonality. To limit heterogeneity of cell banks and ensure consistent product quality, proof of monoclonality has become an important measure of regulatory-approved antibody manufacturing processes. Genetic barcoding offers the inclusion of a cell-intrinsic nucleotide marker which can be repetitively used to validate monoclonality and identity at any given stage and time of a given antibody producer cell clone throughout the production process. Similar approaches based on NGS-analysis of single nucleotide variants or targeted locus amplification products have been published recently^29,30^. However, we speculated that the assessment of genetic barcodes at a pre-defined stable locus offers higher sensitivity, *i.e.* detection of minor subpopulations below 1%, and is not subject to change during cultivation of clonal cell lines.

We cross-validated two monoclonal cell lines by image detection at single cell cloning stage and subsequent Sanger sequencing of barcodes at day 18 (Supplementary Fig. 2a–c). To test the sensitivity of barcode detection within our workflow we mixed the two validated barcoded cell clones at different ratios and measured barcode occurrence via deep sequencing (> 36 × 10^6^ reads). To discriminate genuine barcodes from background introduced by sequencing errors, we included an unbiased knee-point filter method and detected clonal cross-contamination reliable in mixtures at ratios of 1:10–1:1000 (Supplementary Fig. 3a). In addition, we could detect 3, 5, and 17 different monoclonal cell lines in a defined pool (Supplementary Fig. 3b).

**Figure 3:**
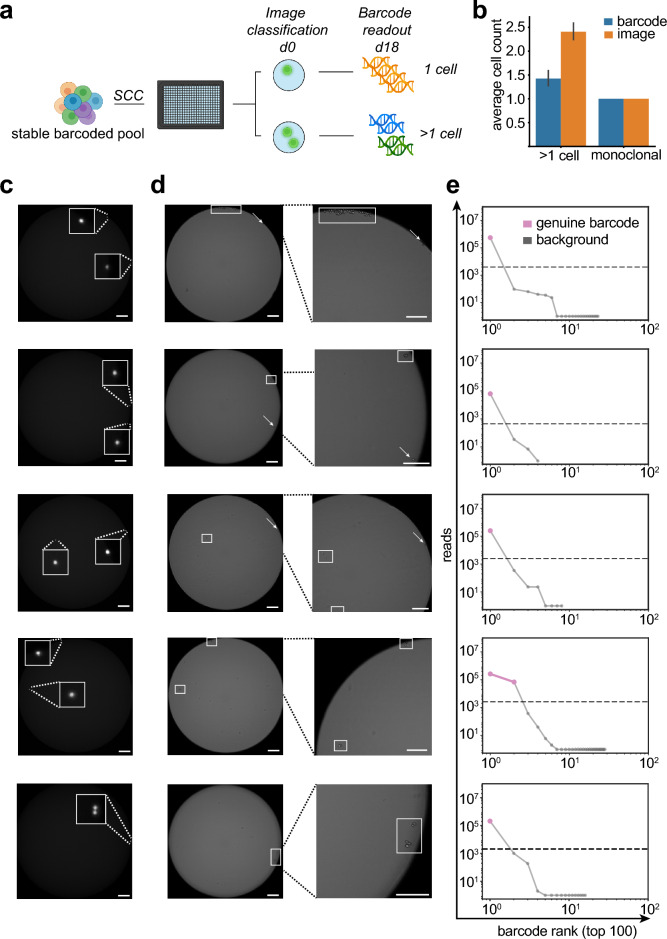
Cellular barcoding can reliably detect clonal status of cell lines during single-cell cloning (**a**) Stable barcoded expression pools were single cell cloned by limited dilution in 384 well plates. Monoclonality was assessed by fluorescent imaging directly after seeding at d0 and barcodes were detected via amplicon deep sequencing at day 18. Wells were grouped based on the initial image based classification in either wells with 1 cell or > 1 cell. (**b**) Bar graphs depicting average number of barcodes detected by the barcoding method as compared to an automated image analysis method. Samples are grouped according to initial image classification to wells containing only 1 cell and > 1 cell, n = 96. Error bars indicate SD. (**c**) Fluorescence imaging at d0 directly after seeding of barcoded stable pools in 384 well plates. This image was used for initial classification of wells. (**d**) Bright-field imaging at day 2 after single-cell cloning (left panel) and magnified view on the cell colonies (right panel). Cell colonies with visible division are marked with a rectangle, cells without visible division are marked by an arrow. Size bar indicates 200 µm. (**e**) The number of barcodes were detected via amplicon deep sequencing and unique top 100 barcodes are plotted. Dashed line indicates the minimum read count cutoff to discriminate erroneous barcodes from genuine barcodes using an unbiased knee point detection algorithm. (**c**) Initial fluorescent imaging directly after seeding cells into 384-well plates during single-cell cloning. Cells are marked by an arrow (**d**).

Monoclonality is traditionally validated by microscopy after limited dilution to achieve a single cell per well based on Poisson distribution^31^. However, limited dilution (LD) is inherently inefficient with most wells either empty or containing more than one cell. To determine if genetic barcoding can improve the single cell cloning process, we compared the number of clones detected with traditional image detection and manual inspection with the amount of clones detected by cellular barcoding. First, we mimicked a traditional single cell cloning process by limited dilution using a Poisson parameter λ = 0.6 (Fig. 3a). We found that for cells, which were classified as monoclonal by traditional image detection, barcoding confirmed the presence of a single barcode in all observed cases (Fig. 3b). Notably, image detection overestimated the number of clones per well by ~ 60% as compared to barcode detection (Fig. 3b). We hypothesized that the number of clones is overestimated by image detection because of poor outgrowth rates during limited dilution.

Therefore, for wells with 2 cells, we inspected consecutive images of wells on d2 after seeding. Notably, we frequently observed only 1 cell with distinct cell division events (Fig. 3c,d, top 3 panels). In one case, we observed cell divisions of both cells, and another case with a potential cell division event (Fig. 3c,d, bottom 2 panels). In case only a single cell survives and gives rise to a new clonal population we should observe a single genuine barcode. We analyzed the new potential clonal populations by deep sequencing at day 18 after seeding. Indeed, for clones where we previously identified only one cell survivor, only a single genuine barcode was detected in the population (Fig. 3e, top 3 panels). In contrast, we observed that for 2 cell survivors, 2 distinct barcodes were detected (Fig. 3e, bottom 2 panels). The barcode analyses also confirmed the presence of only 1 genuine barcode for the cell division event. Intrigued by the possibility to redefine assurance of clonal derivation by a cell intrinsic feature, we calculated the probability of clonality (PoC) when exchanging imaging evidence with barcode analysis. First, we assessed project-specific survival statistics, which represents the best approximation of PoC in the absence of imaging and method-validation studies (α = 0.372, based on 1552 wells with confluence > 10% at day 18 out of a total of 7767 plated wells) Ref.^31^. Next, based on the known relative frequency of barcodes at the time of limited dilution (Table S2), we estimated a “worst-case” probability for barcode collisions in all cases of an amount of k cells > 1 per well. Finally, this calculated to a PoC of 99.63%, when multiplying the probabilities for an amount of k > 1 cells in one well with the probabilities that: (i) at least two barcodes collide and (ii) both cells survive and form colonies. Collectively, these data indicate that barcode detection not only confirmed results of monoclonality assessment via image detection during limited dilution, but outperforms imaging evidence for assessment of PoC. Notably, imaging evidence overestimates the number of clones because of non-proliferating and duplet cells, while barcoding only counts viable monoclonal populations. Thus, NGS-derived cellular barcode readouts represent an improved imaging-independent monoclonality assessment method for CHO producer cell lines, offering a very high PoC (> 99.5%) by analysis of a cell intrinsic feature and project-specific survival statistics^31^. In addition, our barcode methods enables the option to revisit cell line identity (i.e. exclude clone mix-ups) and integrity (i.e. clone cross-contamination) at any given stage and time during the CLD process.

### Cells originating from individual RMCE events share cellular phenotypes

**Figure 4:**
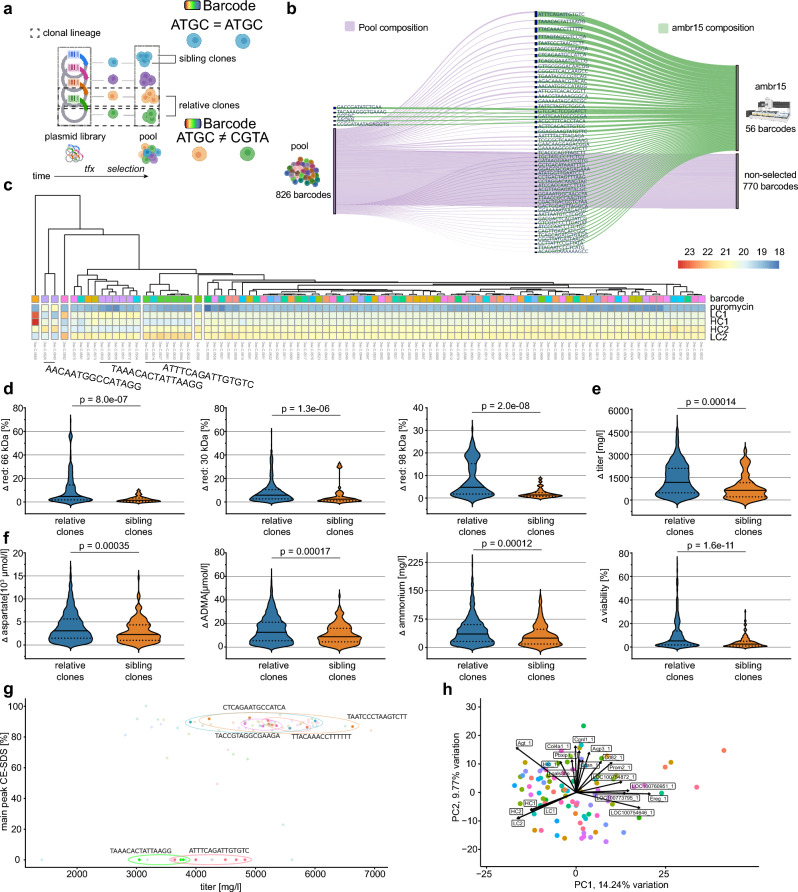
Clonal origin predicts similarity in bioprocess relevant features despite overall similarity (**a**) Experimental outline to evaluate the cellular production performance of clonal cells which originated from different RMCE events. Clones were randomly selected (confluence threshold) and expanded for testing in ambr15 microbioreactors. (**b**) Hierarchical clustering of individual producer clones by antibody chain expression in bulk transcriptome profiling. Note the distance of cells, which share the same barcode. Violin plot comparing (**c**) absolute differences in product quality parameters, (**d**) metabolite concentrations, and (**e**) cellular features between unique barcodes (relative clones) as compared to barcodes with ≥ 3 occurrences (sibling clones). Dotted line indicates the arithmetic mean. FDR-adjusted statistical significance was calculated by Wilcoxon rank-sum test. (**f**) Product quality (main peak measured by CE-SDS) and titer after protein A purification of clonal cells. Clonal cells with identical barcodes are color matched. Barcodes which occurred ≥ 3 times (sibling clones) are highlighted (circle). (**g**) Principal component analysis (PCA) of bulk transcriptome data from 94 randomly selected clonal cell lines. Cells were sampled at day 10 during a 14-day fed batch process in ambr15 bioreactors. Clonal cells with identical barcodes are color matched.

Despite exclusion of position effects and copy number variation by using targeted integration technologies (*e.g.* RMCE-based), cell clones generated from stable expression pools display a relatively high variability of production-relevant readouts such as volumetric titer, metabolite profile and growth rates^15^. The described genetic barcoding method allows us to trace clonal CHO lineages from the time point of transfection onwards. Importantly, this allows discrimination between related cell clones originating from the same TI event but derived from different single cell cloning events (“sibling clones” that share the same barcode sequence and occurred from a cell duplication event in the CHO pool after transfection) and those from different TI events (“relative clones” with different barcodes) (Fig. 4a).

To test whether the phenotypic variability in cell clones is a stochastic event or whether it was predetermined, we generated cell clones from one barcoded CHO producer pool. Cell clones were selected randomly during limited dilution with a confluence threshold of 10% at day 12 in the 96-well plate. The composition of barcodes within all tested clones in the ambr15 stage was comparable to the barcode composition within the originating cell pool (Fig. 4b). Notably, frequent and rare barcode variants (from the original pool) were present in the final clonal populations. Cell clones were then tested for production-relevant markers using a downscale micro bioreactor system (ambr15).

Remarkably, cell clones clustered partially based on antibody chain transcript levels at day 10 of the ambr15 fed batch run (Fig. 4c). We speculated that cells originating from the same TI events (“sibling clones”, same barcode) may show less phenotypic variance as compared to cells from distinct TI events (“relative clones”, unique barcodes). To holistically compare phenotypic distance between clones we next compared pairs of absolute differences within all measured phenotypic data points. We selected 34 “sibling clones” (3 or more barcode occurrences) and 33 “relative clones” and observed a significantly lower variance in the group of “sibling clones” as compared to the group of “relative clones” for secreted antibody chain fragments (Fig. 4d), cellular features (Fig. 4e), and metabolite consumption (Fig. 4f). A list of all tested parameters which were statistically significant is provided (Table S1). The lower phenotypic distance was also apparent when we compared product titer with overall product quality (main peak CE-SDS), where we observed clusters of sibling clones(Fig. 4g)). In agreement with our previous results, bulk transcriptomic profiling during the ambr15 fed batch revealed little overall differences. PCA of gene expression between clones displayed low variation, PC1 explaining 14.24% and PC2 9.77% of variation (Fig. 4h). Notably, antibody chain expression was dominant in the component loading of PC1 and PC2.

The lower variance observed within clones sharing the same barcodes (“sibling clones”) raises the question as to how phenotypic variance is generated within the cell line generation process. A recent study by Weinguny and colleagues hints toward the single cell cloning process, where a distinct DNA methylation pattern emerged in each clone^32^. We therefore asked whether the TI event could influence the epigenetic landscape in a similar way and analyzed the genome-wide methylation profile of 12 clones (6 “sibling clones” same barcode, 6 “relative clones”). Indeed, “sibling clones” cluster closely as compared to “relative clones” (Suppl. Fig. 4a–d). In the analyzed subset most of the differential methylation occurs in intergenic regions and in regions which could not be mapped to defined chromosomes (Suppl. Fig. 4b,c).

Collectively, the data indicate that the majority of observed phenotypic diversity is pre-existing and cell-intrinsic. While some diversity remains within cells sharing the same barcode (“sibling clones”), the majority of phenotypic diversity is explained by the common origin of cells occurring from the identical TI event.

### Clonal diversity control

**Figure 5:**
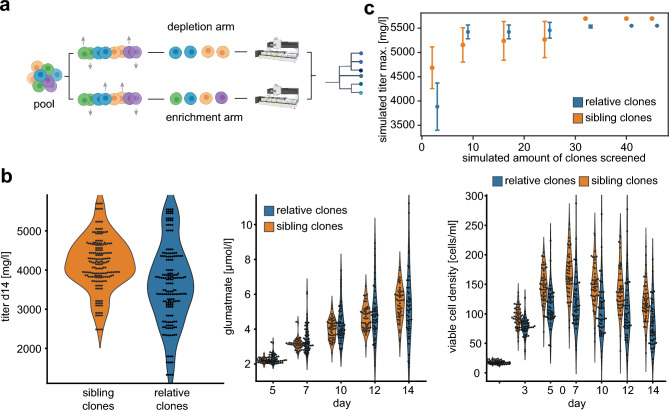
Clonal diversity controls results in leaner CLD process (**a**) Experimental outline of clonal diversity control during the cell line development (CLD) process. Cellular barcodes were used as proxy for cellular diversity and classified in two groups. The depletion arm contained 48 clones with 15 different barcodes, the enrichment arm 48 clones with 48 distinct barcodes. (**b**) Absolute values for antibody titer after protein A purification(left panel), glutamate levels (middle panel), and viable cell density (right panel) grouped by classification into depletion or enrichment arm of cellular diversity. (**c**) Average values for maximum titer simulation of three independent CLD processes when screening different amount of clones. The error bars represent the standard deviations of the simulated titer values.

The increased diversity of cells from distinct TI events implies that we can utilize barcodes as a proxy for cellular diversity during the cell line development process. First, we integrated barcode assessment during hit-picking in the limited dilution process and could therefore monitor the cellular origin of clones during the CLD workflow. Second, we designed one group with enriched diversity, *i.e.* containing only unique barcodes (“relatives clones”), and a second group with decreased diversity, *i.e.* with many “sibling clones” sharing the identical barcode (Fig. 5a). We hypothesized that the group with enriched barcode diversity would show a higher degree of phenotypic variance as compared to the group with decreased barcode diversity.

We evaluated the cellular diversity of cell clones in ambr15 bioreactors during 14 day fed batch production. Intriguingly and in line with our previous results, key phenotypic parameters as volumetric titer values, glutamate consumption and viable cell density did demonstrate substantially increased variability (Fig. 5b). To identify the best performing clone in a population, the screening burden increases with the amount of diversity. Thus, we next simulated the maximum observed titer when systematically sampling different numbers of cell clones. Specifically, we randomly picked n previously measured titer values from cell clones three times independently and plotted the relationship between screening depth (increased amount of n clones) against the maximum titer achieved within each subset. This allowed us to estimate, whether the increased phenotypic diversity would increase or decrease the amount of clones necessary to include the top producer clones.

We observed that despite a lower average titer in the enriched diversity group, the maximum titer was reached when screening substantially lower number of clones and with higher confidence (lower SD) as compared to the decreased diversity arm (Fig. 5c).

Together, our data indicate that cellular barcodes can serve as a proxy for cellular diversity and can improve CLD screening workflows towards enriched diversity of production-relevant parameters substantially.

## Discussion

In this study, we have developed a virus-free method for exhaustive single-copy genomic labeling to track cell populations. In establishing and validating this method, we have focused on a state-of-the-art targeted integration CLD workflow for CHO-cell biofactories producing a panel of three distinct complex antibodies.

Our results show that overall transcriptional diversity within the population is low with no distinct subpopulation present at any time during the CLD process, as previously shown for CHO and HEK cells^33^. In contrast, transgene expression shows a high degree of variability within the host (originally clonal, but passaged over > 6 months) and a stable pool population despite the use of an isogenic, single-copy targeted integration method. Clonal cells, which have undergone recent (< 12 weeks) single cell cloning, display substantially lower intra-population variability in transgene expression. We speculate that the observed variability in transgene expression results from pre-existing and cell-intrinsic factors rather than environmental conditions, supporting the model that no immortalized cell population is uniform over a longer period of time^17^. To test which factors dominantly contribute to diversity in an isogenic targeted integration system, we utilized a genetic barcoding method to trace individual TI lineages after the TI event across stable pool generation, single cell cloning, and subsequent phenotypic characterization. Importantly, moving from previous random integration of transgene towards isogenic targeted integration methods excludes variation due to placement and copy number effects^21^.

In line with previous work^13^, we show that expression cell lines undergo dramatic clonal fluctuations during stable pool selection, with skewed populations already before full population recovery. Additionally, isogenic targeted integration was achieved at the cost of integration efficiency with surprisingly low amount of integration events. We link the low integration efficiency with use of a dual plasmid integration system, which is further reduced by increasing plasmid cargo size^34^ and/or complexity of the expression molecules. While a homogenous population is a desired outcome of a typical engineering approach^21^, remaining diversity enables flexibility and adaptability for efficient cell line development. Our method allows the preservation of remaining diversity for large cargo engineering approaches with inherently low efficiency rates.

Of note, cell lineages which contribute to the highest fraction of the stable pool (barcode ATTTCAGATTGTGTC, Figs. 2e and 4g), result in clones which do express reduced or misfolded protein. We speculate that protein expression utilizes a finite supply of cellular energy, and cells with lower expression burden can divert more resources to growth-supporting processes^35^. This is supported by our previous work where we showed that high producer cell clones devote a substantial proportion (up to 29%) of their global transcriptome towards antibody transgenes^36^. Hence, cells with reduced transgene expression burden quickly dominate the stable population. A simple way to mitigate enrichment of cells with growth advantages over time is the pool separation by single cell cloning at the earliest time point, possibly even during stable pool selection. This will prevent the enrichment of cell clones which found ways to suppress transgene expression even in an isogenic context, possible by CMV promoter methylation^37^.

Assurance of clonal origin, despite growing evidence that clonal origin alone does not guarantee homogeneity^12^, remains a valuable tool to reduce cellular diversity. Direct imaging evidence of single cell origin combined with cell survival statistics displays the preferred option with the highest confidence^31^. The strong focus on the probability of clonality, does however underestimate whether a cell line is of clonal origin, as microtiter wells with more than one cell at the time point of seeding are excluded. We show that NGS-based barcode detection during the single cell cloning process confirms the results obtained by traditional image detection for wells with only 1 cell. Intriguingly, in microtiter wells with > 1 cell NGS-based barcoding can correct false negative wells (with only 1 cell surviving or duplet cells with ongoing cell division) as only surviving cells are evaluated with our method. NGS-based barcoding, by characterizing inherent biological features of the cell line itself, is independent of changes in the single cell cloning workflow that alter the single cell cloning error rate. While populations below 0.1% avoid detection in our project (Supplementary Fig. 3a), NGS-based barcoding exceeds image-based detection which commonly shows error rates between 1–2% (reviewed by Chen, et al.^31^). This technology therefore provides a substantial improved assurance of monoclonality.Further, the method offers the re-evaluation of identity and integrity of cell lines at any later time point as it displays an inherent genetic feature of the cell.

Our results also raise the possibility that drivers for diversity in previous random integration approaches are not necessarily consistent with such drivers in a TI context. Here we show that in a TI context, the clonal origin largely determines phenotypic variability, which in turn is heritable from the original host cell. This suggests cellular diversity as a constant feature, *i.e.* the result of long-term emergence of subpopulations possibly due to genetic and epigenetic adaptations. In contrast, previous studies in the context of random integration postulate that cellular phenotypic variability is linked to environmental influences during single cell cloning^32^, or stochastic gene expression^20^. While we cannot exclude these effects completely in our study, the majority of diversity could be linked to the original cell lineage. The diversity seems however to be, at least partially, driven by pre-existing epigenetic modifications as cells with common origin share genome-wide methylation profiles. The heritage of clonal diversity implies that pre-existing epi-/genetic factors are the main drivers for clonal phenotypic diversity in our setting.

We demonstrated that tracking this diversity allows the increase of phenotypic diversity, which in turn leads to a more efficient screening in simulated CLD rounds. Reduced sampling during clone screening reliably reveals cell clones with high productivity, as titer values quickly plateau with increased screening depth. Consequently, future CLD campaigns may utilize barcode analysis to exclude sibling clones in a revised hitpicking strategy (i.e. expansion of selected cell clones from 384 to 96 well plates). Thus, the freed-up capacity can be utilized by parallel screening of 2–4 CLD campaigns with 48–24 cell clones, respectively. While this will reduce invested resources and screening timelines due to 2–fourfold higher throughput compared to current clone screening protocols (with regard to ambr15 capacity), we further envision very lean screening campaigns, which skip small-scale (ambr15) clone evaluation and directly proceed to scale-up evaluation in ambr250 devices. In summary, genomic barcoding will aid lean CLD screening strategies by providing a novel tool to enrich pre-existing diversity while preserving the benefits of TI, which will ultimately reduce drug manufacturing costs for complex recombinant therapeutic products.

## Conclusions

During the development of a novel engineered cell line various sub-lineages of cell clones occur that exhibit substantial genetic and phenotypic heterogeneity. In the context of TI, we developed a barcoding technology, which allowed us to identify of clonal heritage as the major source of phenotypic variability. Therefore, clonal lineage tracing during cell line engineering displays a new source of inter-clonal heterogeneity monitoring and control with broad implications for biotechnological applications.

## Methods

### Cell culture and single cell cloning

All cell lines were created using a previously generated CHO Host Cell Line (international patent publication number WO 2019/126,634 A2). CHO cells were cultivated in a proprietary chemically-defined medium in 125–500 mL shake flask vessels at 150 rpm, 37 ℃, 80% rH, and 5% CO_2_. Cells can be cultivated in any other chemically defined media after adaptation. Cells were passaged at a seeding density of 3–6 × 10^5^ cells/mL every 3–4 days. Pools of cells that stably express bsAb molecules were generated as previously described by Carver and colleagues^38^. Briefly, expression plasmids were transfected into CHO cells by MaxCyte STX electroporation (MaxCyte, Inc). Transfected cells were then selected and expression of mAb was confirmed by flow cytometry via human IgG staining (BD FACS Canto II flow cytometer, BD). Stable CHO pools were seeded into 384 well plates (seeding density 0.6 cells/well) and expanded randomly to 96 well plates using a confluence threshold of 10%. To generate clonal cell lines, the presence of an individual cell was confirmed by fluorescent and bright field imaging and manual inspectionat day 0 and day 2 after fluorescent staining (NYONE Scientific: SYNENTEC GmbH, Elmshorn, Germany). Cells which showed at least 50% were further expanded and cryoconserved.

### Fed batch production assay

Fed batch production cultures were performed in ambr15 bioreactors (Sartorius AG, Goettingen, Germany) with proprietary chemically defined production media. Cells were seeded at 2 × 10^6^ cells/ml on day 0 of the production stage after adaptation to production media during 2 passages. Cultures received proprietary feed bolus on day 3, 6, 9, and 12. Cells were cultivated for 14 days. Production in the ambr15 system were operated at set points of 37 ℃, dO 40%, pH 7.2, and an agitation rate of 1300 rpm.

### Off-line sample analysis

Process parameters were analyzed with Osmomat auto (Gonotec GmbH, Berlin, Germany) for the measurement of osmolality and a Cedex Bio HT Analyzer (Roche Diagnostics GmbH) for the measurement of product and selected metabolite concentrations. Total cell count, viable cell concentration, and average cell diameter was measured by Cedex HiRes Analyzer (Roche Diagnostics GmbH, Mannheim, Germany). Amino acid and metabolite analysis was performed using an in-house LC–MS (Ultivo Triple Quadrupole LC/MS System, Agilent Technologies Inc., Santa Clara, CA, USA) procedure with stable isotope-labeled internal standards for calibration.

### Generation of barcoded libraries

Constructs used in this study were generated by standard cloning procedures, with sequences synthesized by Twist Biosciences and restriction digest cloning of the final plasmids. The randomized region N15 was introduced into the final plasmid by Genewiz. For genomic DNA, DNA of 10^8^ cells was extracted using the Blood & Cell Culture DNA Maxi Kit (Qiagen) according to manufacturer’s instructions. Amplicons for deep sequencing were generated with primers flanking the barcode region, 100 ng plasmid DNA as input, and 30 cycles of amplification by PCR. For detection of cellular barcodes, 2 µg of gDNA was used as input, with 30 cycles of amplification by PCR with primers flanking the barcode and one primer located outside of the RMCE integration site (to discriminate between off- and on-target integration events). Sequencing libraries were prepared using the KAPA HyperPlus Kit (Roche) using 50–100 ng (fix 20 µl purified PCR) of amplicon DNA as input, no fragmentation step, and between 20 and 24 cycles of amplification of PCR (post-ligation library amplification) to reach 1 µg of total DNA library per sample. Libraries were sequenced by Genewiz using the NovaSeq 6000 platform (Illumina) with 30 M paired-end 150 bp reads per sample.

### Antibody analytics in supernatant

Supernatants were clarified (1000 g, 30 min, 4 ℃ centrifugation and 1.2 μm filtration, AcroPrep 96 Filter Plates, Pall Cooperation). Analytical protein A chromatography was performed by UHPLC with UV detection (Dionex Ultimate 3000 UHPLC fitted with POROS™ A 20 µm Column, Thermo Fisher Scientific Inc.).

Antibody integrity was analyzed after protein A affinity chromatography (PreDictor RoboColumn MabSelect SuRe, Cytiva) and normalization with protein quantitation using UV measurement (Nanoquant Infinite M200, Tecan). Percentage of correctly assembled antibodies (main peak) was assessed by CE-SDS (HT Antibody Analysis 200 assay on the LabChip GXII system, PerkinElmer) under non-reducing conditions by relative quantification of the expected protein size to total protein content.

### Bulk RNA-seq sample preparation and data analysis

Barcoded cells (1 × 10^6^) sampled from the ambr15 bioreactor on day 10 were washed twice in PBS and snap-frozen in liquid nitrogen. RNA extraction, Illumina stranded TruSeq RNA library preparation, poly(A) enrichment, and sequencing (NextSeq, v2.5, high.output 1*75 bp) was performed by Microsynth AG (Belgach, Switzerland). Sequences for the transgene and mitochondrial DNA were included manually into the reference genome (GCF_003668045.3, PICRH1.0). Reads were aligned using the hisat2 package (version 2.2.1)^39^ and transcript abundance was calculated with featureCounts (version 2.0.1)^40^. For downstream analysis we used PCAtools (v2.2.0)^41^ and for differential expression edgeR (v3.32.1)^42^.

### Barcode analysis

To characterize the diversity of the barcode libraries, forward and reverse paired-end raw reads (2 × 150 bp) were trimmed for universal Illumina adapters using cutadapt (v4.1)^43^ and subsequently merged with flash (v1.2.11)^44^. Barcodes were extracted with detection of the flanking region (**M1**: GCTTAGCCGCTTAAT AACATCTAATGCGTA, **M2**: CTTAGCCGCTTAAT AACTTAGCTCGCGTA, **M3**: GCTTAGCCGCTTAAT AACCTCGCTTGCGTA) and all reads which did not match the expected barcode length of 15 discarded. Reverse complement reads were reversed with FASTX toolkit (v0.0.14). Final barcode diversity was estimated using the Chao1 capture-recapture estimator^45^ based on barcodes observed in replicate resampling at varying depths. Collision probability (defined as the fraction of cells at start of experiment which share a barcode due to coincidence of independent barcoding events, rather than common clonal origin) was analyzed as previously described by Horns and colleagues^46^. Quickly, for a given number of cells N, we sampled N barcodes without replacement from the observed barcode pool (with sampling probability proportional to the barcode’s abundance). We calculated the fraction of the sampled barcodes that were unique within the sample, designated p, then the collision probability was 1–p.

### Sensitivity of barcodes as clone cross-contamination reporter

Previously characterized barcoded CHO cell lines (verified as monoclonal by fluorescent microscopy followed by barcode Sanger sequencing) were cultivated and 10^6^ cells were mixed in predetermined ratios. Sequencing libraries were prepared from genomic DNA as described above. Reads were preprocessed as described above with an additional step of barcode clustering using a Levenshtein distance of 1 with Starcode (v1.4)^47^. The number of clone barcodes was detected with an N = 2 for cross-contamination, or N as indicated in Figure S2, using an unbiased knee point threshold based on the read count distribution^46^.

### Single-cell RNA-seq and data analysis

Cells were thawed simultaneously to prevent bias based on different cell age. Cryopreserved cells frozen in exponential growth phase were subjected to sequencing. Single-cell library preparation and sequencing was performed on the 10 × Genomics platform by GENEWIZ Germany GmbH (Leipzig, Germany). Sequences for the transgene and mitochondrial DNA were included manually into the reference genome (GCF_003668045.3, PICRH1.0). Reads were aligned to this custom reference genome and quantified using CellRanger (v6.0.1)^48^. For downstream analysis we used Seurat (v5.0.0)^49^. Cells which contained less than 4000 features or displayed mitochondrial DNA content of more than 5% were discarded. Cell cycle phase was predicted using homologous genes between Mus musculus and Cricetulus griseus for regressing out cell cycle effects^50^. After pre-processing, the 3 datasets were merged into a single Seurat object (FastMNNIntegration method, consistent good performance across datasets)^51^. 5.10 Probability of clonality including cell population distributions.

To estimate the probability of at least two identical clones occurring in a single well, we utilized a Poisson distribution model.

$${P}_{\lambda }\left(k\right)= \frac{{\lambda }^{k}}{k!}{e}^{-\lambda }$$

The parameter λ represents the average number of cells per well and *k* represents the specific number of cells in a well. Clone probabilities ${P}_{i}$ were derived from the relative barcode distributions at the time of limited dilution and normalized such that the sum of all ${P}_{i}=1$. The probability $P\left(K=k\right)$ that $k$ cells are in a well follows a Poisson distribution:

$$P\left(K=k\right)= \frac{{\lambda }^{k}}{k!}{e}^{-\lambda }$$

where $K$ is the random variable for the number of cells in a well. The probability that all $k$ cells are different clones is given by:

$$ P{(} all different {|} k ) = \mathop \prod \limits_{i = 0}^{k - 1} \left( {1{ }{-}{ }P_{0} } \right), $$

where $n$ is the number of different clones and ${P}_{0}$ is the normalized probability of the clone with the highest appearance. It assumes that each cell has the highest probability of being the same clone. Using the highest clone probability for all cells represents the worst-case scenario because it maximizes the likelihood of having at least two identical clones in a well. This approach provides a conservative estimate, ensuring robustness in the analysis. The probability that at least two identical clones are present among $k$ cells is:

$$P\left( at least two identical \right| k )=1-P\left( all different \right| k )$$

The probability that a well with $k$ cells shows cell growth is used as described in “Method 3” by Chen and colleagues^31^ and given by:

$${G}_{k}= \left\{\begin{array}{c} a if k=1\\ k*a*{\left(1-a\right)}^{k-1} if k>1\end{array}\right.$$

where $a$ is the single cell recovery rate, calculated by solving for $a$ in the equation

$$N*\sum_{k=1}^{100}{P}_{\mu }\left(k\right)*\left(1-{\left(1-a\right)}^{k}\right)=W$$

where $N$ is the total number of wells, $W$ is the number of wells with cell growth (defined here by > 10% confluence on day 18 after limited dilution), and $\mu$ is the average number of cells per well. $a$ represents the probability that a single cell will recover and grow into a colony. For wells with more than one cell, the probability of growth is adjusted to account for the possibility that only one cell recovers while the others do not. The overall probability that at least two identical clones occur in a well is calculated by summing over all possible $k$ (from 2 to a maximum $k$, here 10, as more than 10 cells per well are sufficiently unlikely):

$$P\left(at least two identical\right)= \sum_{k=2}^{10}\left(\frac{{\lambda }^{k}{e}^{-\lambda }}{k!}*\left(1-\prod_{i=0}^{k-1}\left(1-{P}_{0}\right)\right)*{G}_{k}\right) $$

This formula describes the probability that at least two identical clones occur in a well, based on the Poisson distribution of cell counts, the normalized clone probabilities, and the cell recovery rate.

### Supplementary Information

Supplementary Figures.

Supplementary Table S1.

Supplementary Table S2.

## Data Availability

Nucleic acids and cell lines encoding for antibody sequences are proprietary to Roche.

## Data availability

The data supporting the findings of this study are available within the article and its supplementary materials. Nucleic acid sequences encoding for antibody sequences are proprietary to Roche.
