# Supplementary material for: Genomic barcoding for clonal diversity monitoring and control in cell-based complex antibody production
Source: Sci Rep. 2024 Jun 25;14:14587. doi: 10.1038/s41598-024-65323-7 (PMC11199663; doi:10.1038/s41598-024-65323-7)
Supplement: Supplementary file 1 — Supplementary Figures. [file 41598_2024_65323_MOESM1_ESM.docx]

**Supplementary Information**

Genomic barcoding for clonal diversity monitoring and control in cell-based complex antibody production

Niels Bauer^1,2^, Christoph Oberist^1^, Michaela Poth^1^, Julian Stingele^2^, Oliver Popp^1^ & Simon Ausländer^1,*^

*^1^Large Molecule Research, Roche Pharma Research and Early Development (pRED), Roche Innovation Center Munich, Penzberg, Germany;
^2^Gene Center and Department of Biochemistry, Ludwig-Maximilians-University Munich, 81377 Munich, Germany.* **Corresponding author: E-Mail:* [*simon.auslaender@roche.com*](mailto:simon.auslaender@roche.com)

**Supplementary Figure 1 | Plasmid barcode libraries**


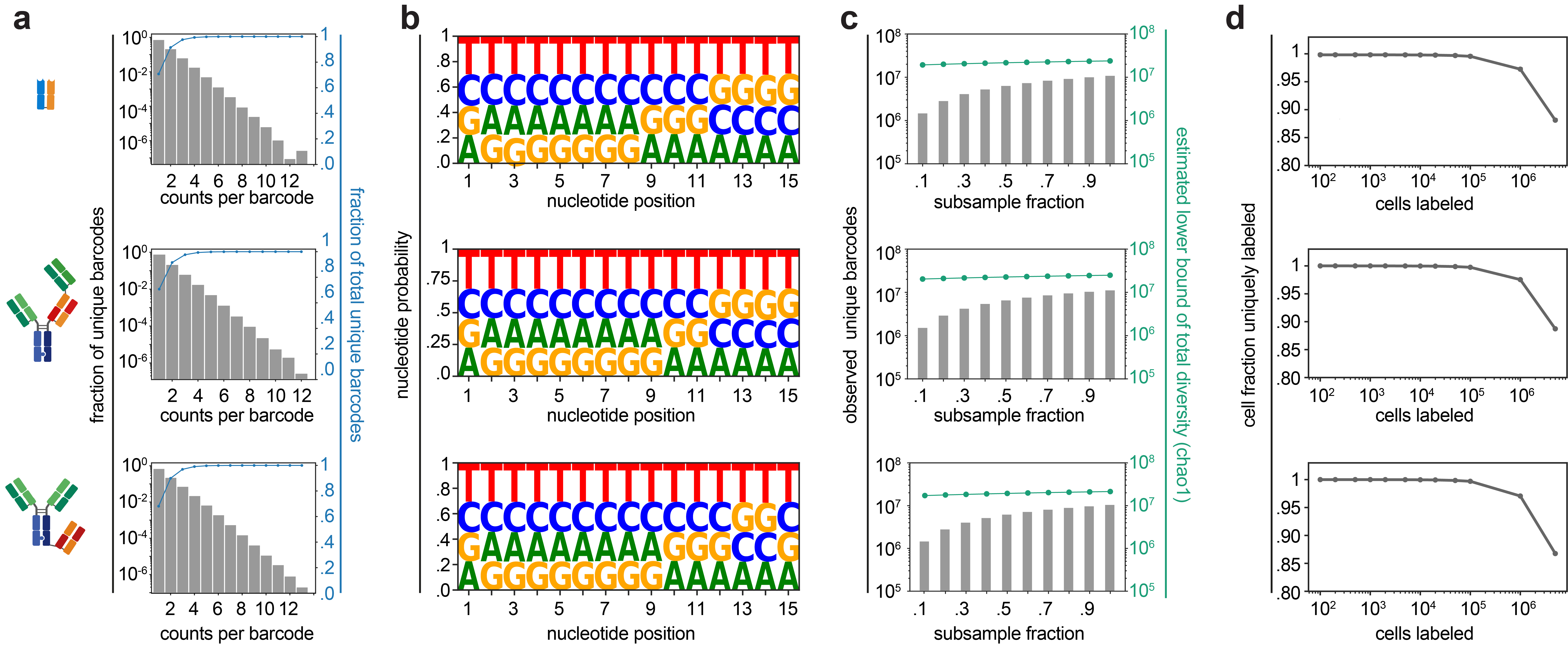


**a)** Diversity of Back plasmid barcode libraries was assessed by amplicon deep sequencing. **b)** Nucleotide probability at each position of the N15 barcode region. **c)** Lower bound of total barcode diversity within each library was estimated based on observed counts using the Chao1 capture-recapture estimator. **d)** Estimation of labeling capacity for each library was estimated by coincidental barcode collisions. Mean values for 100 replicate simulations (resampling).

**Supplementary Figure 2 | Verification of monoclonality for two cell lines used for cross-contamination experiments**


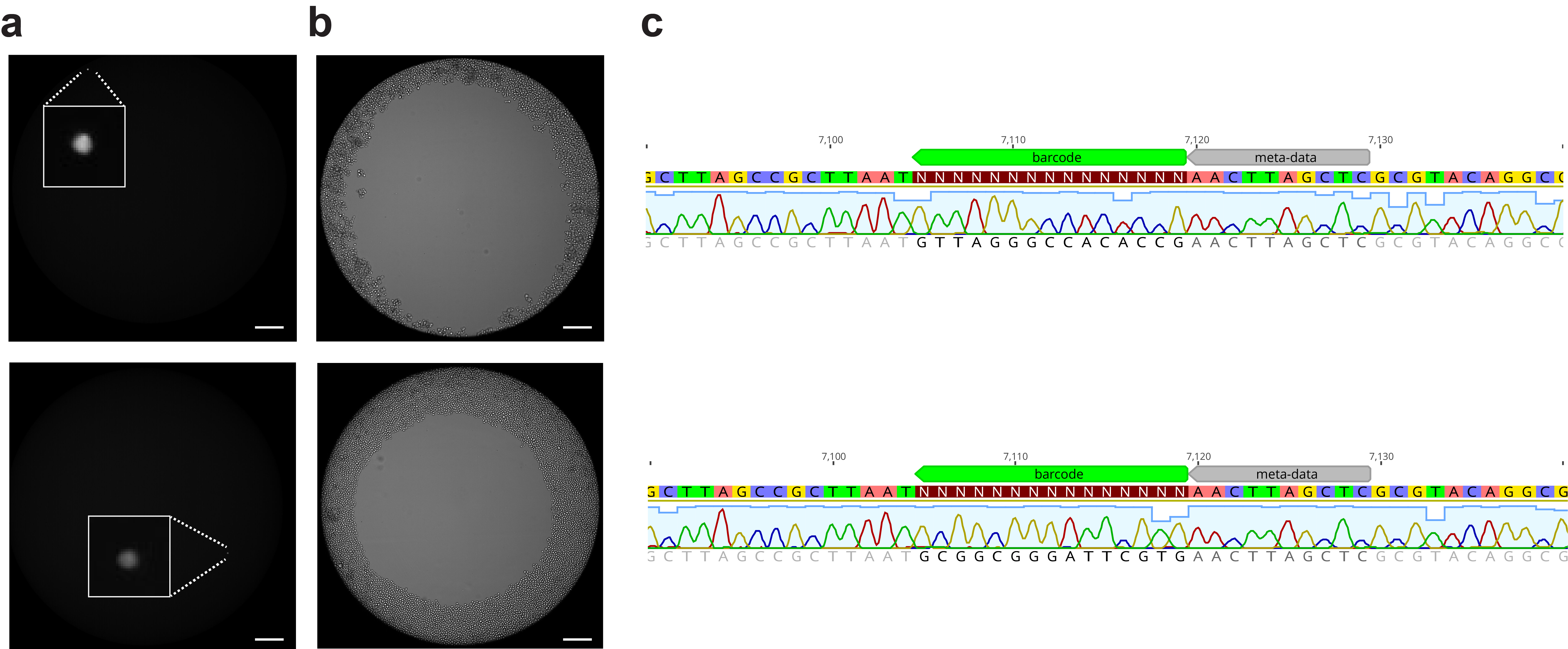


**a)** Fluorescence imaging at d0 directly after seeding of barcoded stable pools in 384 well plates. Image was used for proof-of-monoclonality. **b)** Bright-field imaging at day 12 after single-cell cloning. Scale bars indicate 200 µm. **c)** Sanger sequence trace aligned to the TI locus reference. Generated with Geneious version 2023.1 created by Biomatters.

**Supplementary Figure 3 | Detection of clone cross-contamination**


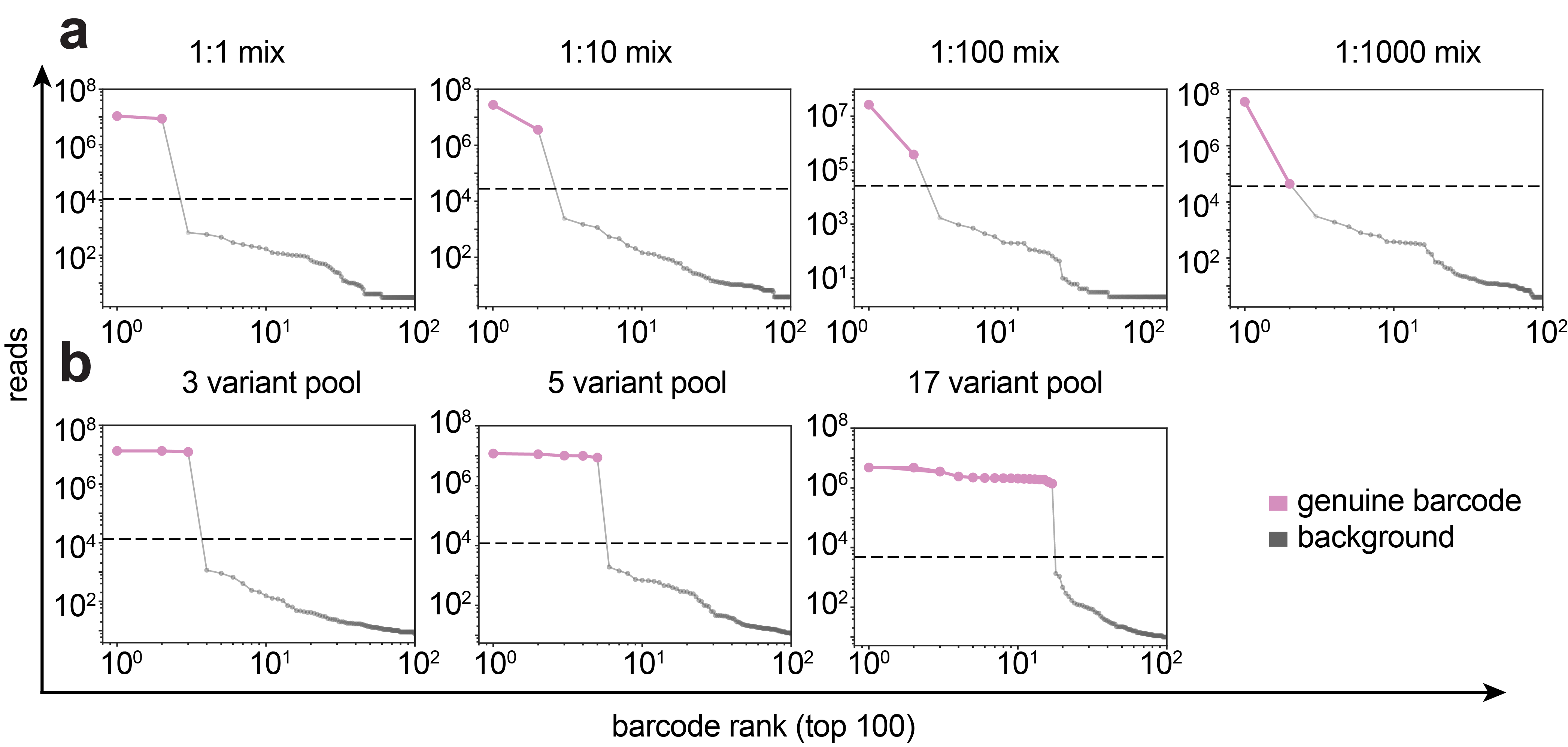


**a)** Two barcoded clones were mixed and barcodes detected via amplicon deep sequencing. **b)** 3, 5, and 17 distinct barcodes were mixed prior to amplicon deep sequencing. Unique top 100 barcodes detected are plotted. Dashed line indicates the minimum read count cutoff to discriminate erroneous barcodes from genuine barcodes using an unbiased knee point detection algorithm.

**Supplementary Figure 4 | Epigenetic profiling shows differential methylation in clones from distinct RMCE events.**


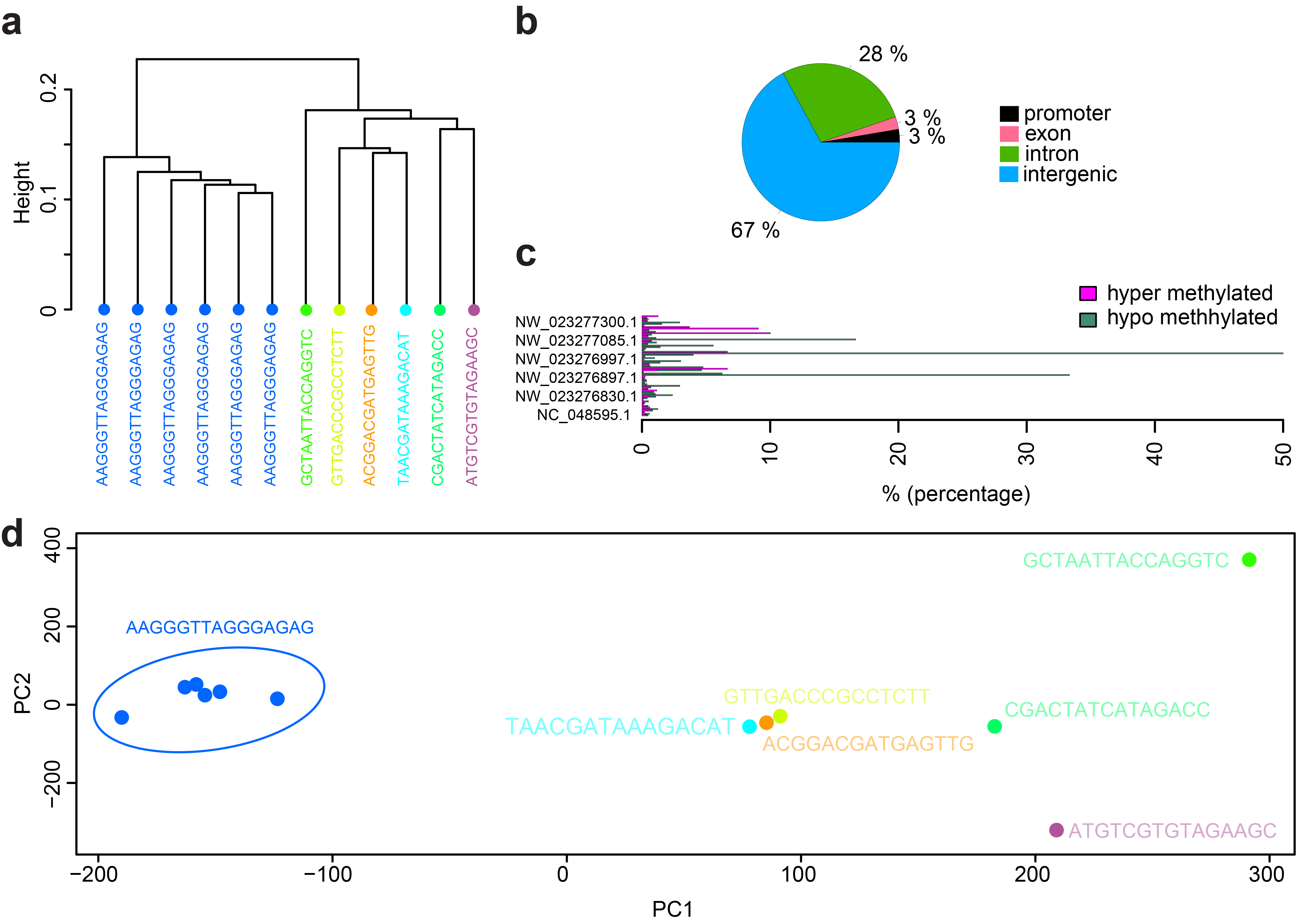


**a)**  Hierarchical clustering of whole-genome methylation analysis for 12 clones, with 6 sharing an identical barcode (blue) and 6 with an unique barcode. **b)** The gene region of significant differential methylation events. **c)** Significant (p-value ≤ 0.05) hyper- and hypomethylation events per chromosome between min and max values observed within the 12 clones. **d)** Principal Component Analysis of all 12 methylation profiles showing principal component 1 and principal component 2 for each sample.
